# Attitudes to Conduct Research Among Physicians Specializing in Older Adult Care: Longitudinal Study

**DOI:** 10.1093/geroni/igaf122.1663

**Published:** 2025-12-31

**Authors:** Jenny van der Steen, Anna van Ede, Sytske van Bruggen, Wing H Tong, Annemarie Moll-Jongerius, Victor Chel, Maaike N Scheffers-Barnhoorn, Belinda W C Ommering

**Affiliations:** Leiden University Medical Center, Leiden, Zuid-Holland, Netherlands; Leiden University Medical Center, Leiden, Zuid-Holland, Netherlands; Leiden University Medical Center, The Hague, Zuid-Holland, Netherlands; LUMC, Rotterdam, Zuid-Holland, Netherlands; Leiden University Medical Center, Leiden, Zuid-Holland, Netherlands; Leiden University Medical Center, Leiden, Zuid-Holland, Netherlands; Leiden University Medical Center, Leiden, Zuid-Holland, Netherlands; HU University of Applied Sciences Utrecht, Utrecht, Utrecht, Netherlands

## Abstract

Attitudes regarding conducting research may be shaped by experiences during medical training and residency. Supervised performing of scientific research might motivate physicians to contribute to the evidence base on medical treatment of older people. We conducted a longitudinal study (2019-2024) to assess perception and attitudes towards conducting research of physicians specializing in older adult care (“elderly care medicine”) in the Netherlands who conducted a mandatory training study on pain and discomfort in dementia. Residents completed validated scales for intrinsic and extrinsic motivation, self efficacy and perception of research at the beginning, midway and end of the 3-year program. Random effect models assessed changes across study phases. Separately, residents’ personal reflection reports were analyzed inductively and, subsequently, against a Self Determination Theory framework with qualitative thematic analysis. Of 78 invited residents, 41 (53%) participated. Changes–in particular a decrease of intrinsic motivation scores–were greatest when developing a research question or data analyses, and in the later phases, mostly returned to initial levels. Self efficacy was the most stable. Starting during the pandemic, being native Dutch and little experience was associated with lower scores. The qualitative results indicated that many appreciated collaborative working in itself as educational. Further, intrinsic motivation for research appeared positively associated with perceived autonomy, competence and relatedness. Both types of data suggest that attitudes regarding doing research are shaped mostly before residency, factors such as a feeling of being in control and relatedness to the research may be used to encourage development of positive attitudes towards conducting scientific research.

